# Takotsubo Cardiomyopathy: A Brief Review

**DOI:** 10.25122/jml-2018-0067

**Published:** 2020

**Authors:** Hilman Zulkifli Amin, Lukman Zulkifli Amin, Ariel Pradipta

**Affiliations:** Indonesian Medical Education and Research Institute, Faculty of Medicine, Universitas Indonesia, Jakarta, Indonesia

**Keywords:** Takotsubo, cardiomyopathy, reversible

## Abstract

Takotsubo cardiomyopathy is a reversible cardiomyopathy with a unique morphological feature of the left ventricle characterized by an apical ballooning appearance known for approximately known 25 years. Catecholamine drive plays an essential role in the pathogenesis and pathophysiology of Takotsubo cardiomyopathy; hence, it is also called stress cardiomyopathy. Physical stress could also have an impact and leads to a greater variety of characteristics in Takotsubo cardiomyopathy.

Supportive and symptomatic medication remains the mainstay therapy with priority to improving the function of the left ventricle for several days and full recovery in 3-4 weeks. Due to its similarity with myocardial infarction, Takotsubo cardiomyopathy requires careful diagnosis and management for the best possible outcome.

## Introduction

Takotsubo cardiomyopathy (TC) is defined by a temporary and reversible systolic abnormality of the left ventricle's apical area resembling myocardial infarction (MI) in the nonexistence of coronary artery disease (CAD) [1]. This clinical entity was initially described approximately 25 years ago [2]. The word “Takotsubo” is a container used by the Japanese to catch octopus, which has a circular bottom and narrow neck, which resembles the heart's condition in TC to a certain degree [3]. There are various types of left ventricular (LV) function abnormalities within this disease [4]. The prevalence is 1.0-2.5%, with most cases to occur in post-menopausal women [3,5]. Many conditions have been linked to TC, like over-stimulation of the sympathetic system, microvascular and myocardial tissue metabolism abnormality, and coronary artery vasospasm [3]. Despite frequently being underdiagnosed, complete understanding is needed to optimize the management of the disease. This review will briefly explain the main features of TC, including definition and management protocol.

## Materials and Methods

Various papers from Pubmed in relation to Takotsubo cardiomyopathy were thoroughly selected and appraised. The results from those papers are discussed and summarized to complete the current review paper.

### Definition and Diagnosis

The well-accepted TC diagnosis criteria is from Mayo Clinic and consists of four components: 1) temporary hypokinesis, dyskinesis or akinesis in LV segments with or without apical involvement; aberration in regional wall motion exceeding past a single vascular distribution; the existence of stress elicitation; 2) the lack of significant coronary artery disease; 3) recent changes detected in the electrocardiogram (ECG) (ST-segment elevation and/or T-wave inversion) or significant elevation of serum cardiac troponins; and 4) non-existence of pheochromocytoma or myocarditis [6]. The summary of the diagnosis criteria for TC is shown in table 1. Usage of diagnostic modalities combinations such as ECG, cardiac biomarkers, echocardiography, coronary angiography, and cardiac magnetic resonance (CMR) imaging will add value to a more precise way in diagnosing TC. Mostly, ECG shows recent abnormalities resembling ACS like ST-segment elevation, especially in the anterior leads (56%) and T-wave inversion (39%). Other forms of ECG abnormalities that may also appear are QT-prolongation, ventricular tachycardia (VT), ventricular fibrillation (VF), and torsade de pointes [7]. Furthermore, a study by Kosuge et al. found that the combination of ST-segment depression in aVR and the absence of ST-segment elevation in V1 could reveal TC with 91% sensitivity, 96% specificity, and 95% predictive accuracy [8]. In addition, as shown by other studies, in order to distinguish between anterior MI and TC, ECG should reveal no reciprocal changes and Q waves with the ST-elevation ratio in leads V4-6 to V1-3 > 1, and also the absence of ST-depression or following inferior ST elevation [9].

**Table 1:** Summary of TC diagnosis criteria [6].

1. Temporary hypokinesis, dyskinesis, or akinesis in LV segments with or without apical involvement; aberration in regional wall motion exceeding past a single vascular distribution; the existence of stress elicitation.
2. No presence of significant coronary artery disease.
3. Recent changes in electrocardiography (ECG) (ST segment elevation and/or T-wave inversion) or significant elevation of cardiac troponin serum levels.
4. Non-existence of pheochromyctoma or myocarditis

In-line with ECG findings, TC also shows an elevated level of cardiac biomarkers showing myocardial disturbance [10]. In 90% of patients, the troponin levels are elevated, often mistakenly diagnosed as ACS [11]. Nevertheless, contradictive to ACS, the highest level of troponin mostly would be <1ng/ml. B-type natriuretic peptide (BNP) and N-terminal pro-BNP (NT-proBNP) have also been found to be frequently increased up to 3-4-fold higher compared to patients with ACS [12]. From one study, significantly elevated levels of these biomarkers were not related to pulmonary congestion or pulmonary capillary wedge pressure, but associated with reduced ejection fraction (EF) and elevated plasma catecholamine levels, hence revealing TC pathogenesis and its severity [12].

The pathognomonic finding of TC during echocardiography is apical ballooning involving LV. This unique morphology was reported to appear in 75% of patients [2]. In 25% of patients, the morphology was reported to follow a mid-ventricular ballooning pattern due to mid-LV akinesis, with no disturbance of apical and basal contraction [13-14]. Furthermore, an impaired LVEF with typical systolic anterior motion (SAM) could also be found within this case. To provide more significant evidence of TC, CMR is an important imaging investigation. CMR could show particular imaging characteristics like right ventricular (RV) involvement and differentiate it from other cardiomyopathies [15]. However, due to difficulties in distinguishing between TC and ACS, coronary angiography could demonstrate a critical role in diagnosing TC. Coronary angiography could more accurately prove normal coronary artery or non-significant atherosclerosis. In addition, a myocardial biopsy could also be performed if there are no contraindications, mostly to show interstitial infiltrates with mononuclear lymphocytes, leukocytes, macrophages, myocardial fibrosis, and contraction bands. Inflammatory reaction and contraction bands show different features in TC and MI and it may reveal coagulation necrosis in the case of coronary artery obstruction [16].

### Cause, pathophysiology and mechanisms

The precise cause, pathogenesis, and pathophysiology of TC are still uncertain. Many hypotheses have been linked with the occurrence of TC. Recently, the most accepted theories are catecholamine-induced cardiotoxicity and microvascular dysfunction, in addition to the complex and integration of neuroendocrine physiology, eventually involving the cognitive centers of the brain and hypothalamic-pituitary-adrenal axis [17,18].

**Figure 1:**
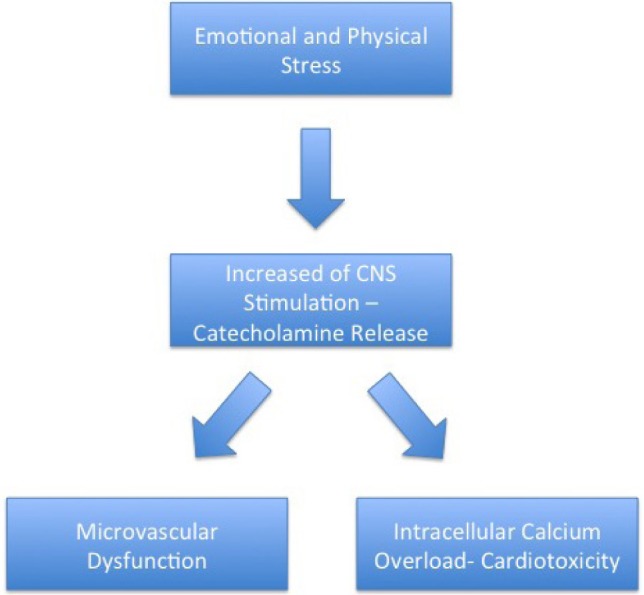
Mechanism of Takotsubo Cardiomyopathy.

A study by Wittstein et al. revealed that the plasma levels of epinephrine were critically elevated in TC patients, with emotional stress as its major precipitating factor. In addition, the study also indicated that the serum catecholamine concentration was two to three folds higher in TC than MI patients [19]. Moreover, other studies also substantiate the catecholamine theory further through exogenously administered catecholamine and pheochromocytoma, resulting in similar features of TC [20-21]. Excessive levels of catecholamines released by the sympathetic nervous system caused by a stressful condition could result in intracellular calcium overload and cardiac dysfunction through b(1)- adrenoreceptor signal transduction pathway (Figure 1) [4]. Calcium overload in myocardial cells consequently leads to ventricular dysfunction and catecholamine cardiotoxicity [22]. Conditions with high catecholamine levels also affected the b(2)- adrenoreceptor resulting in myocyte injury because of calcium leakage due to hyperphosphorylation of the ryanodine receptor [23]. Nevertheless, cardiotoxicity caused significant changes in myocardial features with contraction band necrosis, inflammatory cell infiltration, and fibrosis [24].

It is important to note that a recent body of evidence also revealed that there is a higher prevalence of TC due to physical triggers than that of an emotional trigger. In addition, it was generally agreed that the absence of an isolated trigger should not exclude the diagnosis of this disorder. Due to a large number of possible causes that remain unknown until now, TC may manifest a wide variety of features.

Patients with TC also constantly demonstrated microvascular dysfunction features [26]. These features include the impairment of endothelium-dependent vasodilatation, excessive vasoconstriction, and abnormality of myocardial perfusion (Figure 1) [27]. A study by Uchida et al. revealed that thorough endothelial cell apoptosis was shown by myocardial biopsy [28].

Risk factors of TC include estrogen deficiency, emotional or physical stress, and genetic factors. Most of the patients with TC are postmenopausal women. A study conducted by Ueyama et al. reported that rats who were subjected to stressful conditions and then underwent ovariectomy demonstrated lower LV function than rats with estradiol supplementation [29]. In addition, estrogen may intensify the transcription of cardioprotective factors such as heat shock protein and atrial natriuretic peptide, hence defend from cardiotoxic elements such as catecholamines, calcium overload, and oxidative stress [29-30].

Emotional stress is also playing a major role as a precipitating factor in the occurrence of TC. Stress promotes the response of the sympathetic system, which can be linked to the occurrence of TC [31]. Genetic factors also demonstrated a possible role for TC occurrence. One study revealed that patients with TC have L41Q polymorphism of the G protein-coupled receptor (GRK5) more often than the control group [32]. L41Q polymorphism of GRK5 reacts to catecholamine stimulation and diminishes the reaction of b-adrenergic receptors. In addition, Mediterranean and Asian women have a higher susceptibility to this dysfunction [33-34].

In more detail, several emotional or psychological stressors have been known to initiate the onset of TC, and the structures that mediate these responses are found in central and autonomic nervous systems [35]. The stressors cause brain activation, elevate cortisol, and catecholamine bioavailability. Both circulating epinephrine and norepinephrine released from adrenal medullary chromaffin cells, and norepinephrine released locally from sympathetic nerve endings are significantly increased in the acute phase of TC. This process, which has a functional counterpart of transient apical left ventricular ballooning, initiates myocardial damage through several mechanisms, which are direct catecholamine toxicity, adrenoceptor-mediated damage, epicardial and microvascular coronary vasoconstriction and/or spasm, and elevated cardiac workload. In addition, other risk factors, such as estrogen deprivation, may have a facilitating role, possibly through endothelial dysfunction, as mentioned previously [35].

### Clinical characteristics

Most common clinical characteristics of patients with TC are chest pain and dyspnea [3]. One study revealed that chest pain was present in 185 of 273 patients (67.8%, 95% CI: 62.0-73.0%; range: 20-94.7%) and dyspnea in 40 of 225 patients (17.8%, 95% CI: 13.3-23.3%; range: 4.5-55.5%). More critical clinical presentations like cardiogenic shock (4.2% (95% CI: 2.4-7.4%)) and ventricular fibrillation (VF) (1.5% (95% CI: 0.65-3.9%)) can also be identified [3]. The clinical characteristics are similar with CAD, so diagnostic approach to this clinical entity needs to be done meticulously.

### Treatment and prognosis

Due to its resemblance to MI, first management should focus on the treatment of CAD. Hence, one of the diagnostic criteria of TC is theexclusion of CAD. Therefore, initial therapy includes oxygen inhalation, intravenous heparin, aspirin, and b-blockers [36]. After excluding CAD and further confirmation of TC, aspirin can be stopped. In TC, b-blocker usage is reasonable due to possible high catecholamine state. However, its usage should be avoided when coronary vasospasm is suspected on first presentation [36]. In addition, angiotensin-converting enzyme inhibitor (ACE-I) and angiotensin receptor blocker (ARB) could also be used as part of regional wall motion abnormality (RWMA) management. Furthermore, anticoagulation therapy should be continued even after TC diagnosis confirmation. This therapy is useful to prevent LV apical thrombosis and possible embolic events [36].

However, when patients with TC come to the hospital in the acute phase, supportive and symptomatic treatment should be given. Hemodynamically unstable patients may need cardiopulmonary support, continuous venovenous hemofiltration, and intra-aortic balloon pump [37-39]. Other supporting therapies like diuretics and nitroglycerin may show benefit, since 20% of patients with TC have congestive heart failure (CHF) as a complication [19, 40].

The in-hospital mortality rates varied from 0-8% with recurrence rate range from 0-15% [13, 40-44]. Patients with TC have great prognosis, the recovery rate being 96% [41]. The LV function may begin to recover in several days and fully recuperates in 3-4 weeks [4]. Last but not least, even though therapy guidelines for TC have yet to be arranged, the majority of patients were treated with antithrombotic and heart failure medication for up to twelve months in one of the most recent studies on the subject. Left ventricular function and myocardial edema recovered rapidly within the first two months, with the outcome analysis showing a low bleeding rate and a high short-term survival. Hence, antithrombotic and heart failure therapy might bring significantly benefits in TC management [45].

## Conclusion

TC is a transient and reversible cardiomyopathy with good prognosis. The hallmark feature of TC is apical ballooning in LV similar in its outlook with the so called ‘Takotsubo’, which is a pot for octopus fishing used in Japan. Due to its similar features to MI, a careful diagnosis and management should be performed. Catecholamine levels play a vital role in pathogenesis and pathophysiology of TC, hence it is also called stress cardiomyopathy. TC risk factors include estrogen deficiency, emotional and physical stress, and genetic factors. The mainstay therapy is supportive treatment and is reported to be effective as TC patients’ LV function generally begins to restore in several days and fully recuperates in 3-4 weeks.

## Conflict of Interest

The authors confirm that there are no conflicts of interest.
